# The impact of price transparency and competition on hospital costs: a research on all-payer claims databases

**DOI:** 10.1186/s12913-022-08711-x

**Published:** 2022-11-05

**Authors:** Ahreum Han, Keon-Hyung Lee, Jongsun Park

**Affiliations:** 1 Department of Health Care Administration, Trinity University, San Antonio, TX 78212 USA; 2 Askew School of Public Administration and Policy, Florida State University, Tallahassee, FL 32306 USA; 3 Department of Public Administration, Gachon University, Seongnam, South Korea

**Keywords:** Healthcare price transparency, Hospital cost, Public reporting, Market competition, All-payer claims databases (APCDs)

## Abstract

**Background:**

Public reporting has been considered effective in reducing health care costs by mitigating information asymmetry in the market as payers have incorporated publicly available information mandates into pay-for-performance programs and value-based purchasing. Therefore, hospitals have faced increasing pressures to provide price transparency. Despite the widespread promotion of healthcare transparency, the effectiveness of public reporting has not yet been sufficiently understood. This study analyzed the impact of transparency policy and competition on hospital costs by taking the state operations of all-payer claims databases (APCDs) as a case of interest.

**Methods:**

We employed a fixed-effects regression, which allows the generation of hospital-specific effects, in accordance with the suggestion by the Hausman test. The study samples comprise nonprofit and for-profit general acute care hospitals in the United States for 2011–2017. The finalized dataset ranges from 3547 observations in 2011 to 3405 observations in 2015 after removing missing values.

**Results:**

We found that hospitals in the states with APCDs tend to bear higher average operating expenses than those without APCDs, which may indicate that states maintaining higher healthcare expenditures are more attentive to a price transparency initiative and tend to adopt APCDs. With regard to competition, the results showed that weak market competition is significantly associated with higher operating costs, supporting the traditional competition theory. However, the combined effect of APCDs and competition did not indicate a significant association with operating expenses. Further investigation showed a continued tendency for a weak intensity of competition to be linked to lower hospital operating costs in states without APCDs. For those located in non-APCD adopted states, market consolidation helped hospitals coordinate care more effectively, economize operating costs, and enjoy economies of scale due to their large size. Similar trends did not appear in APCD-adopted states except for in 2015.

**Conclusions:**

This study observed limited evidence of the impact of APCDs and market competition. Our findings suggest that states need to make multifaceted efforts to contain hospital costs, not solely depending on the rollout of cost information or market competition. Market concentration may lead to coordinated care or cost economization in some cases. Still, the existing literature also demonstrates some potentially harmful impacts of increased concentration in the healthcare market, such as inefficient use of resources, unilateral market power, and deterrence of innovation. The introduction of a price transparency tool may require additional policy actions that take market competition into consideration.

**Supplementary Information:**

The online version contains supplementary material available at 10.1186/s12913-022-08711-x.

## Background

The U.S. is considered one of the most advanced nations in terms of medical technology and practice [1]. It is also known as a country that bears the highest healthcare expenditure per capita in the world. U.S. healthcare spending reached $3.8 trillion in 2019, which amounted to 17.7% of its gross domestic product [2]. A substantial amount of U.S. healthcare spending has financially burdened federal and state governments as well as employers and individual households. Despite the astronomical expenditure, the U.S. healthcare system has not achieved the *Triple Aim*, defined as “improving the individual experience of care; improving the health of populations; and reducing the per capita costs of care for populations” [3]. A plausible remedy for this problem may reside primarily in reducing healthcare costs, because access and quality are impeded mainly by the unbearably high costs and their opaqueness [4].

Public reporting has been regarded as effective in reducing health care costs by mitigating information asymmetry in the market [3, 5, 6]. Payers have incorporated publicly available information mandates into pay-for-performance programs and value-based purchasing [7, 8]. Empowered by public data profiling, health plans may opt to shift patient volume to health care providers that provide better quality and more cost-efficient care [9]. Hospitals face increasing pressure to provide price transparency. Since the Affordable Care Act (ACA) of 2010, price transparency has been widely promoted across states [10]. The CMS Hospital Price Transparency final rule (84 FR 65,524) mandates that hospitals make all price information publicly available online including standard charges for items and services for all payers and 300 stoppable services, effective as of January 1, 2021. Accordingly, the CMS is seeking to increase monetary penalties for noncompliance with the final rule accordingly [11].

A number of states have taken a series of market-based approaches when reforming their healthcare systems. Among many market-friendly approaches, public reporting has been considered one of the effective instruments for curtailing skyrocketing healthcare costs. Some states had preexisting price transparency rules and policies in place prior to the implementation of federal mandates. Some of these efforts are publicly funded, while others are operated by nonprofit organizations, community collaboratives, or large purchasers of health care. The New York State Department of Health has collected and disseminated information regarding outcomes of risk-adjusted coronary artery bypass graft (CABG) mortality rates in 1991. In 2009, California began to report hospital-acquired infections to provide incentives for hospitals to secure patient safety. Leapfrog Group for Patient Safety, a nonprofit watchdog organization, is another major public reporting actor in the health care sector.

All-payer claims databases (APCDs) is another public reporting scheme that has aggregated state-wide healthcare data from various payers since 2007. The unique virtue of the APCDs initiative, as its name “all-payer claims” implies, resides in the fact that it strives to collects “claims data” as opposed to “billed charges.” Also, the database encompasses many different kinds of healthcare payers, such as medical and pharmacy claims, physician and hospital files, and dental claims, not to mention Medicare and Medicaid. To alleviate information asymmetry in the health care market, various efforts to develop these databases across states have accomplished this goal to a certain extent, in that more than half (27 out of 50) of the states have adopted the policy either legislatively, or through voluntary efforts by various stakeholders. A comprehensive list of APCD-related state activities is presented in Appendix 1.

Despite the widespread promotion of healthcare transparency, the effectiveness of public reporting has not yet been sufficiently understood. Contradictory findings in the literature warrant examination to determine whether transparency policy contributes to cost reduction in the health care market. Advocates of public reporting believe that public release of performance data encourages improvement in health care delivery and assists consumers in making better-informed decisions about their health care [5, 6, 12–14]. Transparency may spur competition and eventually curtail costs through two major pathways in healthcare: by providers, and by consumers. Competition is essential in motivating providers to achieve better value for their money through controlling costs, promoting innovation, enhancing quality, and maximizing efficiency [15]. From the providers’ perspective, performance data will not only inform purchasers when selecting insurance plans, but also motivate providers to outperform in order to protect their reputations and the demand for their services [13, 16]. Transparent market information is also expected to be useful for monitoring competitors’ strategy and performance.

There are many reasons to believe that health care consumers can foster competition under mandated transparency. Given that a paucity of price information has been a historical hinderance for healthcare consumers, they are likely to use publicly available information to price shop before receiving treatment [17, 18]. It will be particularly useful for non-emergency medical services, such as diagnostic imaging, without compromising quality [19]. Empirical evidence has demonstrated that patients’ use of price information was linked to lower total payments for common medical services [20]. Transparent pricing will greatly benefit those with high deductible health plans (HDHPs) by allowing them to compare prices to minimize their out-of-pocket expenditures [21]. Public reporting may be particularly useful for uninsured and self-paying patients because hospitals tend to bill them higher fees for the same services for which most health insurers pay less [22–24].

In contrast, scholars argue that price transparency may lead to increased healthcare prices by reducing competition, enabling provider collusion, and making prices more uniform [25]. Enhanced transparency may drive price increases if goods are imperfect substitutes [26]. Specifically, transparency may increase the demand for healthcare services as consumers become better informed. The increased demand may override the competition effect of transparency. It is reported that high-performing hospitals in New York raised their prices immediately after data reporting, because high performing surgeons had to accommodate the growing demand for their services with limited resources [27]. Additionally, costs may be rationally viewed as a good proxy for quality [10]. If patients perceive that high costs are associated with high quality, health care providers are unlikely to economize their costs.

State adoption of APCDs can serve as a useful proxy for transparent climates because the databases collect comprehensive payment data and offer stakeholders access to comparable price information [17]. However, disclosing that large-scale healthcare data has often been politically and technically challenged [28]. Thus, the program necessitates a proper assessment of its effectiveness in the wake of growing demand for healthcare information as well as an assessment of the barriers to accessible healthcare information databases. If APCDs are effectively utilized toward those goals, more efficient healthcare may be achieved, maximizing value with higher-quality, and lower-cost healthcare choices. In this context, this study investigates whether APCDs are significantly associated with lower hospital costs. In addition, we intend to investigate the role of competition in addressing cost challenges. Market competition is included in our model as a variable of interest, because public reporting and competition are considered complementary based on traditional economic theory and, in fact, transparency policy draws upon the theory of competition [29–31]. Thus, this study will also explore the effects of competition, which are presumably spurred by making health information publicly available.

## Methods

### Data

The study samples comprise nonprofit and for-profit general acute care hospitals in the United States for 2011–2017. The finalized dataset ranges from 3547 observations in 2011 to 3405 observations in 2015 after removing missing values. There are three main data sources: hospital cost reports published by the U.S. Centers for Medicare & Medicaid Services (CMS), the American Hospital Association (AHA)’s annual hospital survey data, and the official website of the APCD Council. The data on AHA subsidiaries’ lobbying efforts in each state were collected from the Center for Responsive Politics’ website. Table 1 shows a description of the variables and their sources.

**Table 1:** Variable description and sources

Category	Variable	Description	Data sources
Hospital cost	Expenses (E)	Annual total operating costs of hospitals (logged)	Medicare cost report
Transparency	APCD (A_1_)	An indicator of whether states have adopted APCDs (adopted = 1, otherwise = 0)	The APCD Councils
Competition	HHI (C)	The sum of squared market shares of hospitals in a Hospital Referral Region (HRR)	AHA
Hospital characteristics	Size (S)	The number of staffed beds	
	Urban (U)	Location of hospital (Urban = 1, otherwise = 0)	
	Nonprofit (O)	Ownership of hospital (nonprofit = 1, otherwise = 0)	
	Teaching (T_1_)	The hospital is affiliated with medical schools (Teaching = 1, otherwise = 0)	
	Adjusted admission (A_2_)	All patient care activities, including inpatient and outpatient services, undertaken in a hospital	
State characteristics	Lobbying (L)	The total amount of money that AHA subsidiaries spent on lobbying efforts in a state (logged)	The center for responsive politics

### Model specification

The impact of the APCDs on healthcare expenditures is conceptualized as a cross-sectional time-series model. The method used for the investigation is fixed-effects regression, which allows the generation of hospital-specific effects, in accordance with the suggestion by the Hausman test. The specification of the model for this study is illustrated as follows:

$${E}_{it}=f\left(A,C,S,U,O,{T}_{1},{A}_{2},L,{T}_{2}\right)+{h}_{i}+{e}_{it}$$

where:

- E = total operating expenses per adjusted admission for hospital i in year t (logged)
- A_1_ = whether a state has adopted APCDs in year t
- C = the sum of the squared market shares of hospitals in an HRR (market competition around hospital i)
- S = the number of beds at hospital i in year t (hospital size)
- U = urbanity of hospital i (urban = 1, otherwise = 0)
- O = ownership of hospital i (non-profit = 1, for-profit = 0)
- T_1_ = whether hospital i is affiliated with a medical school (teaching = 1, non-teaching = 0)
- A_2_ = the total number of adjusted admissions in hospital i
- L = amount of lobbying dollars for an AHA subsidiary
- T_2_ = a vector of time dummies
- h_i_ = hospital fixed effects that control for overall trends in adoption, and
- e_it_ = the error that is i.i.d. (0, s^2^)

### Construction of the variables

Hospital operating expenses. Total hospital operating expenses are of such vital concern that most governments want to control these through health policy channels. There has been a wide variation in operating costs even after adjusting for the varying complexity of patient needs, as patient are treated by each hospital at different regional wage levels. This difference indicates that thousands of dollars in additional expenses could have been saved if there had been an effective cost reduction regulation for those hospitals with higher expense structures. Total operating expenses means the operating expenses incurred as part of the delivery of care, including expenses on capital related costs, employee benefits, administrative and general expenses, housekeeping, dietary, cafeteria and so on. The operating expenses are transformed to logarithms to mitigate skewness in the dollar term.

APCDs adoption*.* Adoption and duration times were used as variables of interest to estimate the impact of the policy adoption itself as well as the history of policy adoption on hospital costs. States that enacted APCDs through legislative statute as well as states with voluntary reporting initiatives (mostly led by nonprofit organizations) are considered adopted and are coded as “adoption = 1.” Since the importance of informal networks for knowledge creation has been increasingly acknowledged, these voluntary efforts may be as influential as formal regulations [32]. Strong interests are treated as “adoption = 0,” as they have not adopted APCDs yet.

Market competition*.* The Herfindahl–Hirschman Index (HHI) is the standard measure used by the Department of Justice and the Federal Trade Commission to estimate the extent to which the market is concentrated and is frequently used by health care researchers. HHIs are calculated as the sum of the squared market shares of hospitals in a market; a lower value of HHI represents a more competitive market. In theory, markets with more comparable firms are inclined to be more competitive than those that are monopolistic [33]. Therefore, the magnitude of competition is inversely associated with this variable. HHIs are calculated as the sum of the squared market shares of hospitals in a hospital referral region (HRR), where a lower value of HHI represents a more competitive market. Market shares are calculated by dividing the number of beds in a hospital by the sum of beds in the HRR to which the hospital belongs.

Lobbying efforts*.* The interest group model of regulation finds that legislations are regarded as devices thorough which interest groups gain market power [34]. Interest groups like the AHA take advantage of specialized knowledge to frame legislation in favor of their interests [4]. Furthermore, those special interest groups are likely to affect the dynamics of competition by lobbying the Legislature to pass laws to limit competition [33, 34]. For instance, the AHA sought to support state Certificate of Need laws to restrict the expansion of freestanding ambulatory surgical centers [34]. In this sense, the amount of dollars spent on lobbying at the state level was included to control political influence on policy adoption and competition.

Characteristics of hospitals. The variables associated with the characteristics of individual hospitals are included to control for hospital specific characteristics of the samples. We used hospital size, which was determined by calculating the total number of beds, as larger hospitals may reduce costs in production as the result of economies-of-scale. If hospitals are located in urban areas, are nonprofit, or are teaching affiliated, they are coded as “urban = 1,” “non-profit = 1,” or “teaching = 1” respectively, otherwise they are coded as 0. Adjusted admissions include all patient care activities undertaken in a hospital such as inpatient and outpatient services.

## Results

### Descriptive analysis results

The total operating costs of hospitals per adjusted admission across the states during 2011–2017 were examined. As in Table 2, hospital numbers ranged from 3,405 in 2015 to 3,547 in 2011 due to missing or erroneous values in the data. The annual average expenses of hospitals per adjusted admission were $10,496 during the seven-year range. This number gradually increased from $9,673 in 2011 to $11,383 in 2017.

**Table 2:** Descriptive statistics (*N* = 24,191)

Variables / Year	2011	2012			2013		2014	2015		2016	2017
*Continuous Variables*											
Number of hospitals	3,547	3,488		3,484			3,421		3,405	3,425	3,421
Operating expenses ($ mil)	158.01	171.79		178.06			186.31		197.49	208.91	218.66
HHI	.0229	.0237		.0239			.0244		.0247	.0246	.0246
Size (#)	179	181		181			182		182	182	182
Lobbying ($)	65,647	62,064		58,959			71,001		70,433	65,757	91,484
Adjusted admissions (#)	15,498	16,003		15,966			16,260		16,828	17,285	17,608
*Dichotomous variables*											
Adoption (#)	16 (32%)	18 (36%)	20 (40%)			22 (44%)		22 (44%)		22 (44%)	23 (46%)
Urban (#)	529 (14.9%)	508 (14.6%)	492 (14.1%)			465 (13.6%)		467 (13.7%)		462 (13.5%)	460 (13.4%)
Nonprofit (#)	2,740 (77.2%)	2,706 (77.6%)	2,722 (78.1%)			2,694 (78.7%)		2,685 (78.9%)		2,697 (78.7%)	2,723 (79.6%)
Teaching affiliation (#)	877 (24.7%)	876 (25.1%)	883 (25.3%)			896 (26.2%)		927 (27.2%)		924 (27.0%)	915 (26.7%)

There is no dramatic change in the number of states that adopted the policy legislatively. In 2011, the 16 states that implemented the APCDs, either mandatorily or voluntarily, included: Kansas, Maine, Maryland, Massachusetts, Michigan, Minnesota, New Hampshire, Oklahoma, Oregon, Rhode Island, Tennessee, Utah, Vermont, Virginia, Washington, and Wisconsin. Colorado and Connecticut successfully enforced the APCD law, and Arkansas and New York followed suit in 2013 and in 2014 respectively. In 2017, Delaware, Florida, and Washington newly mandated APCDs, while Tennessee put its APCD operation on hold. As for the duration, Kansas, Maine, New Hampshire, and Vermont are the states that have had a tradition of APCDs for over 10 years, as of 2018. Maryland has the longest history (20 years) among the states having APCDs.

In terms of market competition, the healthcare market became less competitive as shown by the increasing tendency of HHI values. Given that a lower competition index means higher hospital market competition (or lower concentration), hospital market structures are more likely to be consolidated over time. The size of hospitals, measured by the number of beds, remains about the same, or 181 on average, and 14% of the hospitals in the sample are located in the 100 largest U.S. metropolitan areas. Over three quarters of the hospitals are nonprofit, while approximately one quarter of the hospitals are affiliated with a medical school. The amount of lobbying dollars spent by AHA subsidiaries grew from $139,439 at minimum in 2013 to $213,469 at maximum in 2017. The AHA’s lobbying activity tends to be more prevalent as time passes. The amount spent by each state differs greatly from zero to over one million dollars.

### Regression analysis results

Before conducting a panel data analysis, a Hausman specification test was carried out to determine the suitable model between fixed and random effects. The test result rejects the null hypothesis, being in favor of the fixed-effects regression. In theory, fixed-effects estimation is appropriate when we analyze a population as a whole, not a fraction of it, and the case is applicable to this study. Table 3 reports the results of the estimated regression model for hospital total operating costs. We separated the samples and constructed two additional models, one with the operation of APCDs (Model 2) and one without the operation of APCDs (Model 3).

**Table 3:** The estimated regression models for hospital cost

	(1) All	(2) With APCD	(3) Without APCD
APCD adoption	0.0877***		
	(0.00499)		
HHI	0.200***	0.00916	0.178**
	(0.0727)	(0.203)	(0.0745)
APCD*HHI	0.0485		
	(0.0650)		
HHI*2012		-0.141	-0.000946
		(0.112)	(0.0417)
HHI*2013		-0.129	-0.0496
		(0.108)	(0.0424)
HHI*2014		-0.171	-0.0977**
		(0.108)	(0.0429)
HHI*2015		-0.185*	-0.0996**
		(0.109)	(0.0430)
HHI*2016		-0.160	-0.153***
		(0.109)	(0.0427)
HHI*2017		-0.176	-0.115**
		(0.111)	(0.0447)
Size	0.000423***	0.000810***	0.000748***
	(4.48e-05)	(7.30e-05)	(4.80e-05)
Urban	0.00196	-0.0220	0.00552
	(0.0201)	(0.0491)	(0.0242)
Nonprofit	0.0283**	0.0117	0.0619***
	(0.0114)	(0.0243)	(0.0109)
Teaching	-0.0503***	0.0196	-0.00880
	(0.0173)	(0.0265)	(0.0183)
Lobby	0.205***	0.0857**	-0.0387**
	(0.0156)	(0.0432)	(0.0161)
Adjusted admission	16.75***	7.137***	12.79***
	(0.381)	(0.592)	(0.428)
2012		0.0762***	0.0611***
		(0.00708)	(0.00351)
2013		0.109***	0.0943***
		(0.00684)	(0.00369)
2014		0.148***	0.127***
		(0.00658)	(0.00380)
2015		0.193***	0.160***
		(0.00660)	(0.00382)
2016		0.239***	0.210***
		(0.00661)	(0.00385)
2017		0.275***	0.246***
		(0.00696)	(0.00407)
Constant	17.84***	18.00***	17.70***
	(0.0127)	(0.0276)	(0.0131)
Observations	24,191	6,297	17,894
R-squared	0.146	0.399	0.369
Number of hospitals	3,835	1,314	3,337

^***^p < 0.01, ^**^p < 0.05, ^*^p < 0.1 and standard errors in parentheses

In the model, adoption status is positively and statistically significantly associated with hospital costs. Hospitals in the states with APCDs tend to bear higher average operating expenses than those without APCDs, holding others equal. Contrary to what we expected, the result shows that competition is not statistically significant when associated with hospital costs. It can be interpreted that APCD deployment alone is not as effective in cutting costs as states hoped.

Additionally, this may indicate that states bearing higher healthcare expenditures are more attentive to a price transparency initiative and tend to adopt APCDs in comparison with those states with lower healthcare costs. Based on the results regarding the time effects in Models 2 and 3, greater increases in costs were identified in APCD-adopted states than in states without APCDs throughout the observed periods (2012–2017). It is logical to assume that some states have used APCDs as a strategy to mitigate an increasing healthcare expenditure burden, such as Medicaid spending [35].

With regard to competition, the results showed that weak market competition is significantly associated with higher operating costs, supporting traditional competition theory. However, the combined effect of APCDs and competition did not indicate a significant association with operating costs. Further examination was conducted to explore the relationship between APCDs, the competitiveness of the market, and the time effect, because the policy may interact with other factors.

When competition and time trends are considered together, the coefficients of competition have been significant and negative since 2014 in Model 3. It indicates a continued tendency for a weak intensity of competition to be linked to lower hospital operating costs in states without APCDs. For those located in non-APCD adopted states, market consolidation helped hospitals coordinate care more effectively, economize operating costs [36], and enjoy economies of scales through their large size [37, 38].

It is noteworthy that similar trends did not appear in APCD adopted states (Model 2) except for in 2015. The largely insignificant association between competition and costs illustrates that market competition has been unrelated to cost containment efforts in comparison with the base year of 2011. Given the trend of increasing concentration of health care providers (Fulton, 2017), we can reason that declined market competitiveness may have potentially driven some states to adopt APCDs in order to cope with the expected increase [35], but the transparency tool alone was not able to deliver the intended consequences.

In terms of hospital characteristics, all the control variables except for urbanity were shown to be statistically significant. It is found that larger hospitals tend to bear higher operating costs. The positive and statistically significant relationship between ownership and cost implies that nonprofit hospitals had higher operating expenses than their for-profit counterparts. It is mainly due to the fact that nonprofit hospitals tend to be larger than for-profit hospitals. Additionally, it has been identified that hospitals located in states that are under more influence from interest groups maintain higher operating costs. The significant association with the degree of lobbying efforts and higher hospital costs infers that those states may encounter political challenges in implementing transparency tools in healthcare.

## Discussion

The possible pathways for understanding how transparency works can be found in the theories upon which this research relies. First, institutional change in favor of price information transparency may well alter an organization’s governance structure [39]. To survive in a more transparent market, healthcare providers are expected to economize costs and boost efficiency [10, 16]. Furthermore, transparency may hold providers more accountable for the costs they charge by avoiding opportunistic behavior [40]. APCDs may also significantly curtail agency costs in the healthcare market by alleviating information asymmetry between physicians and patients especially in states where competition mechanisms are in place [41, 42]. The expectation that patients will price shop could incentivize hospitals to cut costs.

This study observed limited evidence of the impact of APCDs and market competition. Based on our findings, we suggest that governments need to make multifaceted efforts to contain hospital costs, not solely depending on either rollout of cost information or market competition. Market concentration may lead to coordinated care or cost economization in some cases. However, the existing literature demonstrates some potentially harmful impacts of increased concentration in the healthcare market, such as inefficient use of resources, unilateral exercise of market power, and deterrence of innovation [15, 37, 43]. Also, even if cost reductions were induced by mergers, they may not last very long [44].

The healthcare market is becoming less competitive, and the markets for insurance, hospital, and physician services tend to be more consolidated in the U.S. [45, 46]. This is where transparency schemes should come into play. As Gu & Hehenkamp (2014) warned, an unwarranted market structure can wipe away positive competition effects [47]. In the absence of transparency instruments, sizable hospitals are hesitant to behave cost-effectively, and non-price competition may persist. Within this vein of thought, the system for making price and quality information publicly available must be designed with extensive care to circumvent undesirable price escalation [48]. The information should be widely used by patients, regulators, and insurers to price compare, rather than by providers who would use it to tacitly manipulate the market in concert in order to avoid harming their revenues.

The introduction of a price transparency tool may require additional policy actions in consideration of market competition [15]. To improve providers’ performance meaningfully through the policy and competition mechanism, states may also need to devise complementary instruments that assist consumers with price shopping. One major issue that should be simultaneously wrestled with is that many consumers are not willing to use the given healthcare information. One research discovered that only ten percent of consumers actually used the healthcare databases available for them during the first year of the investigation [19]. Transparency policy can boost its usefulness when combined with other instruments that incentivize patients to shop for high-quality and low-cost providers. Consumer outreach and education are central to the policy success because consumers can only be meaningfully empowered when they have easily accessible, user-friendly, and trustworthy information [13, 49].

Our findings should be considered in light of the study’s limitations. The scope of this study was limited to operating costs, even though we acknowledged that quality of care as well as patient satisfaction are also pivotal determinants of the overall soundness of the healthcare delivery system in the U.S. Future scholars may want to assess the policy’s overall impact with quality, distributional concerns, and patient satisfaction in mind. Furthermore, additional micro approaches could be particularly beneficial. A close observation of health consumers’ behavior is crucial in that the policy impact is likely to be corroded unless the revealed information is fully utilized for health choices. Future research may also propose more assistive tools to help consumers better understand and easily compare healthcare services, to eventually foster price competition.

## Conclusion

The evidence in this study shows the limitations of APCDs and market competition. We conclude that the impact of APCD implementation is minimal, and the impact of market concentration varies in different contexts. Adopting a price transparency policy tool was associated with higher operating costs. In non APCD-adopted states, cost reduction was detected along with weakened market competition, while there were few statistically significant predictors of hospital costs for those located in APCD-operated states.

There have been regulative efforts learning toward transparency in healthcare as shown in the recent Executive Order on the improvement of price and quality transparency in order to lower healthcare costs by increasing competition (Executive Order No. 13,877, 2019). The rule mandates that hospitals across the U.S. reveal all standard charges for all hospital services and standard charges for at least 300 ‘shoppable’ services in a consumer-friendly manner. Considering the fact that the number of uninsured individuals increased under the Trump Administration along with escalating cost sharing efforts [e.g., HDHPs and health savings accounts (HSAs)], the significance of transparency should not be underestimated.

It is undeniable that transparency is a prerequisite for the existence of a well-functioning market competition. However, collecting and publicly reporting healthcare information has often led governments to new opportunities and challenges at the same time, because the movements toward transparency have the potential to transform existing healthcare market structures [50]. It would be useful for future research to address the political and technological challenges involved in implementing transparency policy and offer possible solutions to overcome barriers and secure the intended policy impact.

## Supplementary Information

**Additional file 1: Appendix 1.** Status of APCDs Adoption as of 2020.

## Data Availability

The data that support the findings of this study are available from the American Hospital Association but restrictions apply to the availability of these data, which were used under license for the current study, and so are not publicly available. Data are however available from the authors upon reasonable request and with permission of the American Hospital Association.

## Abbreviations

ACA: Affordable Care Act
APCD: All-payer claims database
AHA: American Hospital Association
CMS: Centers for Medicare & Medicaid Services
HHI: Herfindahl–Hirschman Index
HDHP: High-Deductible Health Plan
HRR: Hospital Referral Region
HSA: Health Savings Account
