# Supplementary material for: The impact of price transparency and competition on hospital costs: a research on all-payer claims databases
Source: BMC Health Serv Res. 2022 Nov 5;22:1321. doi: 10.1186/s12913-022-08711-x (PMC9636618; doi:10.1186/s12913-022-08711-x)
Supplement: Supplementary file 1 — Additional file 1: Appendix 1. Status of APCDs Adoption as of 2020. [file 12913_2022_8711_MOESM1_ESM.docx]

**Appendix 1: Status of APCDs Adoption as of 2020 ^[[1]](#footnote-1)^**

| State | Adopted | | | Not Adopted | | First Year Data  Collected |
| --- | --- | --- | --- | --- | --- | --- |
|  | Mandatory | Voluntary | Implementing | Strong Interest | No Activity |  |
|  | 16 | 8 | 3 | 10 | 13 |  |
| Alabama |  |  |  |  | X |  |
| Alaska |  |  |  | X |  |  |
| Arizona |  |  |  |  | X |  |
| Arkansas | X |  |  |  |  | 2013 |
| California |  | X |  |  |  | 2013 |
| Colorado | X |  |  |  |  | 2012 |
| Connecticut | X |  |  |  |  | 2012 |
| Delaware | X |  |  |  |  | 2017 |
| Florida | X |  |  |  |  |  |
| Georgia |  |  |  |  | X |  |
| Hawaii |  |  | X |  |  |  |
| Idaho |  |  |  | X |  |  |
| Illinois |  |  |  |  | X |  |
| Indiana |  |  |  |  | X |  |
| Iowa |  |  |  |  | X |  |
| Kansas | X |  |  |  |  | 2004 |
| Kentucky |  |  |  | X |  |  |
| Louisiana |  |  |  |  | X |  |
| Maine | X |  |  |  |  | 2003 |
| Maryland | X |  |  |  |  | 1998 |
| Massachusetts | X |  |  |  |  | 2009 |
| Michigan |  | X |  |  |  | 2010 |
| Minnesota | X |  |  |  |  | 2009 |
| Mississippi |  |  |  |  | X |  |
| Missouri |  |  |  |  | X |  |
| Montana |  |  |  | X |  |  |
| Nebraska |  |  |  |  | X |  |
| Nevada |  |  |  | X |  |  |
| New Hampshire | X |  |  |  |  | 2005 |
| State | Adopted | | | Not Adopted | | First Year Data  Collected |
|  | Mandatory | Voluntary | Implementing | Strong Interest | No Activity |  |
| New Jersey |  |  |  | X |  |  |
| New Mexico |  |  | X |  |  |  |
| New York | X |  |  |  |  | 2014 |
| North Carolina |  |  |  | X |  |  |
| North Dakota |  |  |  |  | X |  |
| Ohio |  |  |  |  | X |  |
| Oklahoma |  | X |  |  |  | 2011 |
| Oregon | X |  |  |  |  | 2010 |
| Pennsylvania |  |  |  | X |  |  |
| Rhode Island | X |  |  |  |  | 2010 |
| South Carolina |  | X |  |  |  | 2014 |
| South Dakota |  |  |  |  | X |  |
| Tennessee |  |  |  | X |  | 2009 |
| Texas |  | X |  |  |  |  |
| Utah |  | X |  |  |  | 2009 |
| Vermont | X |  |  |  |  | 2007 |
| Virginia |  | X |  |  |  | 2011 |
| Washington | X |  |  |  |  | 2017 |
| West Virginia |  |  | X |  |  |  |
| Wisconsin |  | X |  |  |  | 2006 |
| Wyoming |  |  |  | X |  |  |

1. Source: Authors’ analysis of APCDs’ websites (www.apcdcouncil.org) [↑](#footnote-ref-1)
